# Competencies That Japanese Collegiate Sports Coaches Require for Dual-Career Support for Student Athletes

**DOI:** 10.3390/ijerph191811503

**Published:** 2022-09-13

**Authors:** Goichi Hagiwara, Kayoko Kurita, Shinichi Warisawa, Satori Hachisuka, Jim Ueda, Kensuke Ehara, Katsuhiko Ishikawa, Kosei Inoue, Daisuke Akiyama, Masakatsu Nakada, Masafumi Fujii

**Affiliations:** 1 Department of Sport Science and Health, Kyushu Sangyo University, 2-3-1 Matsukadai, Higashiku, Fukuoka City 813-8503, Fukuoka, Japan; 2 Graduate School of Frontier Sciences, The University of Tokyo, 5-1-5 Kashiwanoha, Kashiwa City 277-8582, Chiba, Japan; 3 Graduate School of Education, The University of Tokyo, 7-3-1 Hongo, Bunkyo-ku, Tokyo 113-8654, Japan; 4 Interfaculty Initiative in Information Studies, The University of Tokyo, 5-1-5 Kashiwanoha, Kashiwa City 277-8582, Chiba, Japan; 5 Faculty of Sociology, Otemon Gakuin University, 2-1-15 Nishiai, Ibaraki City 567-8502, Osaka, Japan; 6 Faculty of Business, Hannan University, 5-4-33 Amamihigashi, Matsubara 580-8502, Osaka, Japan; 7 Office of Management and Planning, Osaka University, 1-1 Yamadaoka, Suita 565-0871, Osaka, Japan; 8 Department of Judo and Kendo, Tokai University, 4-1-1 Kitakaname, Hiratsuka City 259-1292, Kanagawa, Japan; 9 Department of Physical Education, School of Liberal Arts and General Education, National Defense Academy, 1-10-20 Hashirimizu, Yokosuka-City 239-8686, Kanagawa, Japan; 10 Faculty of Sports and Budo Coaching Studies, National Institute of Fitness and Sports in Kanoya, 1 Shiromizu-cho, Kanoya 891-2393, Kagoshima, Japan

**Keywords:** sports, collegiate athletes, higher education, educational development, sports management

## Abstract

The purpose of this study was to clarify coaches’ competencies (COM) regarding dual-career (DC) support for student athletes in Japan. The questionnaire survey was conducted at 31 universities with an online survey URL that was distributed to 300 collegiate athletic coaches. In total, there were 152 respondents (female, 19; male, 133; mean age, 43.15 ± 12.07 years; coaching experience, 12.83 ± 9.72 years). The online survey adopted a Japanese-translated version of the Dual-Career Competency Questionnaire (DCCQ), which has been translated into nine languages and is widely used in European counties. The scale consists of six factors and 33 items, each rated on a five-point scale of importance (IM) and possession (PO). The Japanese version of the DCCQ was validated through a confirmatory factor analysis, and the internal consistency of the items was confirmed by calculating the Cronbach’s alpha coefficient. In addition, we examined differences between the IM and PO for DC support by *t*-tests and by calculating effect sizes. The validity and reliability of the Japanese version of the DCCQ were confirmed based on the goodness-of-fit index and Cronbach’s alpha coefficients, respectively. Our examination of the differences between IM and PO in DC support revealed that coaches perceived the importance of DC support but did not possess the necessary COM to offer DC support. That finding was similar to those of previous European studies. In particular, the Japanese coaches who participated in our online survey recognized the importance of COM in terms of “collaboration with various stakeholders and departments” for DC support but did not feel as though they held sufficient COM in that regard. In other words, the improvement of DC support requires the development of a coaching program that fosters COM to work with various stakeholders. This key insight provides a direction and specific focus for programs to improve coaches’ DC support for student athletes.

## 1. Introduction

The COVID-19 pandemic caused most Japanese universities to close their campuses and conduct classes online, resulting in a situation where many student athletes were unable to pursue a dual career (DC), balancing the application of both academic knowledge and sports. Resultantly, it was reported that, during the COVID-19 pandemic in Japan, many student athletes did not properly develop an identity that balanced academic endeavors and athletics, while many athletes felt a sense of apathy [1]. Hagiwara et al. [1] found that the appropriate development of a student athlete’s identity and a reduction in apathy require social support from significant others. In this context, the Japan Association for University Athletics and Sport (UNIVAS) established the DC division, which comprises researchers, career experts, and Olympic athletes, to support student athletes and develop programs for their healthy DC progress [2]. Dual career, for student athletes in Japan, is “the concept of pursuing athletic success while simultaneously engaging in one’s own career development during the active athletic program” [3]. Student athletes gain a variety of benefits from this, including a balanced lifestyle, good sense of identity, high self-esteem, and sound mental health [4,5]. However, it is very challenging for student athletes to strike a balance where they achieve a good performance in academics and athletics during higher education [6]. A dual career is also a source of psychological stress for student athletes [7]. Finding and maintaining that balance can be challenging and requires an optimal environment and support if they are to continue to successfully develop [8]. Accordingly, the successful DC of student athletes requires the development of a support network with coaches and team staff and the implementation of a DC support program [9]. In particular, the coach plays a central role in socialization for student athletes [10]. Moreover, coaches influence athletes’ self-perceptions and their athletic and non-athletic activities [11,12]. Critically, student athletes’ DCs must be supported by the coaches instructing them in athletics.

In Europe and the United States, identifying the current state of DC support from coaches is a necessary step in determining the optimal DC support strategy for student athletes [13]. However, Ronkainen et al. [14] mentioned that most previous research on student athletes’ DC in higher education focused on athletes’ perceptions and behaviors and rarely addressed the role that coaches could potentially play in the developmental process of athletes. In addition, no studies in Japan have examined coaches’ perceptions of student athletes’ DC support or coaches’ awareness of their role, meaning that we have few insights into the current issues and needs relating to DC support for Japanese student athletes. Thus, we deemed that it was necessary to clarify the current situation regarding DC support for coaches in order to identify the optimal DC support strategies for Japanese student athletes. Previous studies suggested that high-quality DC support from coaches has several positive impacts on student athletes. For example, there are benefits for their sporting performance [15], the smooth transition of student athletes to a high-performance environment [16], and the transition to a career after athletic retirement [17,18]. In particular, high-quality DC support from coaches could provide multiple benefits to athletes in terms of enabling them to effectively balance elite sports and academics [13]. However, there have been no studies in Japan focusing on the specific DC support process and competencies that coaches need to support student athletes’ DCs.

If practitioners understand the competencies required in their particular area or occupation, it leads to their professional development [19,20,21]. In addition, assessments of competencies have been used in clinical psychology [22], social work [23], school counseling [24], and career guidance [20]. In the field of sports science, competency scales used to provide direction and specificity to athlete development and coaching [25,26]. In addition, studies on the assessment of coaches’ competence in terms of DC implementation with student athletes are conducted in European countries. The Dual-Career Competency Questionnaire (DCCQ) consists of 33 competencies divided into six competency factors: (1) advocacy and cooperation, (2) reflection and self-management, (3) organization, (4) awareness of student athletes’ environment, (5) empowerment, and (6) relationships. The DCCQ has been translated into many languages and is widely used in nine European countries: the Netherlands, France, the UK, Spain, Sweden, Belgium, Slovenia, Italy, and Poland. Defruyt et al. [27] examined 288 coaches from nine EU member states to determine the relationship between the six factors of the DCCQ and the competencies that coaches need in order to deal with specific professional challenges. The results indicate that those with good advocacy and cooperation were able to respond effectively in an environment where parents and professors provided little DC support for student athletes. In addition, those with greater empowerment could offer better DC support when student athletes were in a new social environment in which they were separated from their families and living in dormitories. Namely, coaches with greater capabilities for DC support for student athletes were more successfully able to deal with the various challenges faced by student athletes. However, while researchers in European countries have been assessing coaches who provide DC support to student athletes [28], no research has been conducted on coaches’ competencies for DC support, and no assessment scale has been developed in Japan, although the UNIVAS was established to promote DC support for student athletes. Understanding coaches’ competencies regarding DC support leads to more specific student athlete support strategies [26]. Therefore, we aimed to preliminarily develop an assessment scale to identify competencies related to DC support for coaches who work with student athletes. In addition, we assess coaches’ perceptions of DC support and identify the relevant competencies needed by Japanese coaches based on the differences between the importance of DC support competencies as perceived by coaches and students’ retention scores. Furthermore, we examined the associations with coaches’ employment status and years of experience based on the recommendations of a previous study [13].

In summary, we examined the association between coaches’ employment status and years of coaching experience and students’ retention scores to clarify the current status of DC support in Japan. In particular, we implemented this study to identify specific methods of DC support best suited to student athletes in Japan.

## 2. Methods

### 2.1. Participants

The participants consisted of 152 coaches from 31 Japanese universities. They were made up of 66 university faculty members, 30 university staff, and 56 outsourced coaches, 19 of whom were female and 133 of whom were male. The average age of the participants was M = 43.15 ± 12.07 years. The average coaching history was M = 12.83 ± 9.72 years. Table 1 presents the details of the participants’ characteristics.

### 2.2. Procedure

The DCCQ-J was translated into Japanese, and the content and wording were checked for linguistic quality by experts in sports science and education with research experience in English-speaking countries, experts working with the DC Specialist Group at the UNIVAS, and active coaches, including a collegiate coach who was an Olympic gold medalist and had coached a Japanese national team. The study was approved by the Institutional Review Board of the University of Tokyo (no. 22-1). An online survey form was emailed to the head coaches of each team through the sports administration departments of the 31 universities that cooperated in the study. The number of coaches to be surveyed was confirmed in advance with the cooperating universities, resulting in 300 coaches being included in the survey. We adopted the 300 coaches requested for this survey that were affiliated with teams at a level enabling their teams or players to compete in national collegiate championships. An online survey was sent to the 300 coaches, and 152 coaches agreed to respond and fill out the survey form. The response rate was 50.7%. The online survey began with informed consent, explaining the purpose of the survey and the rights of the participants. A link to the online survey was distributed to each university coach. To maximize the response rates, formal (e.g., official email) and informal (e.g., through personal contact or a phone call) reminders were used.

### 2.3. Analysis

In this study, a confirmatory factor analysis (CFA) was first conducted on the importance and possession scales to validate the factor structure of the DCCQ-J. In the CFA, the following goodness-of-fit indices were used: The Root Mean Square Error of Approximation (RMSEA), the Comparative Fit Index (CFI) [29], and the Tucker-Lewis Index (TLI) [30], as in previous studies [13]. The evaluation criteria for the RMSEA were an excellent model fit with a value below 0.06, reasonable fit with a value below 0.08, and poor fit with a value above 0.10. Regarding the CFI and TLI, values above 0.90 indicated an acceptable fit, and those above 0.95 indicated an excellent fit [31,32]. Finally, Cronbach’s alpha was used to measure the factors’ internal consistency: An alpha coefficient greater than 0.70 indicated that an item’s internal consistency was acceptable [33]. We calculated the averages (mean and standard deviation) of the importance and possession of the competencies among the participants. In addition, paired *t*-tests were conducted to examine differences in the perceived importance and possession of competencies, and effect sizes were calculated with Cohen’s *d*. A one-way ANOVA was conducted to examine the association between coaches’ employment status and possession scores. Correlation coefficients were calculated to examine the relationship between the years of coaching and possession scores. All analyses were conducted using IBM SPSS 27.0 and AMOS 27.0 (IBM Corp, Armonk, NY, USA).

### 2.4. Instruments

In this study, we used the Japanese-translated version of the DCCQ (DCCQ-J), which consists of 33 items and six factors, as originally developed by Defruyt et al. [13]. Table 2 shows the English–Japanese correspondence of the 33 items. The scale consists of a five-point rating of the importance (IM) and possession (PO) of DC support. Participants responded to each of the 33 IM and PO questions.

Participants responded to the question, “How important do you think the following competencies are for you as an instructor in supporting student athletes in their dual careers?” using a five-point scale ranging from “1—Not at all important” to “5—Very important”. In addition, regarding their responses to the question, “To what extent do you have the following competencies in supporting dual careers for student athletes?”, respondents were asked to base their answers on a five-point scale ranging from “1—Not at all possessed” to “5—Sufficiently possessed”. The factor structure of the DCCQ-J, as in the original version, consists of six competency elements.

The advocacy and cooperation competencies (ACC) consist of the coaches’ competencies in networking and coordinating with universities, clubs, sports federations, families, and other relevant stakeholders to create a DC environment for student athletes. Secondly, reflection and self-management competencies (RSC) consist of the competencies necessary for coaches themselves to perform self-care and self-monitoring when implementing DC support for student athletes. Third, organizational competencies (OC) consist of competencies that correspond to the organization to which a coach belongs, such as the ability to act in accordance with the mission of the university (educational mission).

Awareness of student athletes’ environment (ASAEC) includes the ability to take a holistic view of a student athlete’s life (not just their athletic performance). Empowerment competencies (EC) are how the coach enhances the student athletes’ competencies, such as improving a student athlete’s ability to plan the management of their athletic and academic balance. Relationship competencies (RC) then consist of the ability to maintain relationships with student athletes, such as the provision of emotional support when they face setbacks.

## 3. Results

### 3.1. Results of Validation of the DCCQ-J Scale Structure

The results of the validation factor analysis indicated that the goodness-of-fit indices for the DCCQ-J were CFI = 0.970, TLI = 0.958, and RMSEA = 0.044 for the scale measuring importance. In addition, on the scale measuring possession, we found that CFI = 0.959, TLI = 0.950, and RMSEA = 0.045. The factor loadings for each competency ranged from 0.45 to 0.86 for the items measuring importance and from 0.46 to 0.88 for the items measuring possession. The details of the factor loadings are reported in Table 3. To evaluate the internal consistency of each factor, Cronbach’s alpha was calculated, and the results indicated that all factors were within the criterion ranges of 0.76–0.90 for the scale measuring importance and 0.77–0.89 for the scale evaluating possession. Cronbach’s alpha for the overall scale was 0.85 for possession and 0.86 for importance. For both scales, all factors were within the criterion range (Table 3). 

### 3.2. Importance and Possession of Perceived Competencies Related to DC Support for Student Athletes

The mean values of the importance and possession of the competencies related to the DC support of the participants were calculated, and the results indicated that all six competencies were perceived as important, especially RSC and RC, which were recognized as the most important competencies (*M* = 4.46, *SD* = 0.52 and *M* = 4.46, *SD* = 0.54, respectively). On the other hand, regarding the degree of possession, the coaches indicated that they perceived five related competencies, excluding ACC (*M* = 2.91, *SD* = 0.61) (Table 4).

The paired-sample *t*-tests showed significant differences between the perceived importance and student retention for all competency factors (*p* < 0.001), and all the means were higher for importance than for retention. The effect sizes were moderate for OC (*d* = 0.50), while ACC (*d* = 1.95), RSC (*d* = 0.89), ASAEC (*d* = 0.80), EC (*d* = 0.94), and RC (*d* = 0.76) had large effect sizes. Table 3 indicates the results of the paired *t*-tests comparing the importance and possession of the perceived competencies.

Table 5 indicates the results of a one-way ANOVA to examine the relationship between the employment status of coaches and their possession scores. In particular, the results for the ACC, ASAEC, and RC indicated that outsourced coaches had significantly lower possession scores than the university faculty did. To examine the relationship between the years of coaching and possession scores, correlation coefficients were calculated, and the results indicated positive correlations between the years of coaching experience and ACC (*r* = 0.33, *p* < 0.01), RSC (*r* = 0.34, *p* < 0.01), OC (*r* = 0.33, *p* < 0.01), ASAEC (*r* = 0.45, *p* < 0.01), EC (*r* = 0.34, *p* < 0.01), and RC (*r* = 0.33, *p* < 0.01) (Table 6).

## 4. Discussion

Coaches play an important role in supporting successful dual careers for student athletes. However, there has been little research focusing on the specific competencies that coaches require to provide high-quality DC support in the Japanese context. Therefore, we developed the DCCQ-J to allow coaches themselves to self-evaluate the importance and their possession of DC support competencies, and we identified the current status of the competencies related to DC support among coaches in Japanese collegiate sports. First, we examined the factor structure of the DCCQ-J using the CFA and were able to confirm its factorial validity. The CFA fit indexes for the six-factor structure of the DCCQ-J were in the acceptable to excellent range, and the internal consistency of each competency factor fulfilled the criterion values. Thus, the DCCQ-J is suitable as a scale for evaluating coaches’ competencies in DC support for student athletes with a certain degree of reliability and validity. 

The quality of dual-career support and the development of qualified support providers have gained attention around the world [34,35,36]. The scale was developed to clarify the DC support status of coaches, and optimal DC support strategies for student athletes are being investigated in European countries [13,29]. In the future, it is expected that the DCCQ-J will be used in further research on DC support strategies for coaches of student athletes in Japan. All competency ratings made by the participants in this study ranged in importance from “very important” to “important”, indicating that coaches recognized the importance of all competencies. In terms of possession scores for the competencies, coaches perceived themselves as “possessing” but not as “fully possessing” five competencies, and the coaches perceived that there was still considerable potential for improvement in their competencies. In addition, the ACC was below average on the five-point scale, indicating that the coaches might have perceived that they “do not possess” the competency. Furthermore, we examined the differences between the importance and possession of all competencies, and coaches might have perceived the competencies they possessed as lower than the competencies they perceived as important for DC support for student athletes. 

In the following section, we discuss each competency with reference to previous research. Relationship competencies were recognized as the most important competencies for coaches in supporting DCs. The difference in the levels of possession was a competency for Japanese coaches to develop to support their student athletes’ DCs. In sport psychology research, a strong relationship based on trust proves an important condition for positive outcomes when working with athletes [37]. In other words, coaches’ interpersonal skills were important for athletes’ development [38]. Similar to the findings of our study, competencies in maintaining relationships with athletes were important for DC supporters in European studies [13,27]. In addition, coaches need to build relationships with student athletes to support holistic DC implementation in Europe and the United States [39]. Hence, it necessary to develop a program in Japan to develop the competencies that coaches need to maintain relationships with student athletes.

Reflection and self-management competencies were another competency that identifies as an important element of DC support. The differences between importance and possession indicated that RSC is a competency that Japanese coaches perceive as necessary to DC support for student athletes. Reflection and self-management competencies confirm the importance of self-evaluating DC support programs in an evidence-based approach to provide the best possible support for student athletes [35]. It is important for coaches to engage in self-reflection and wellbeing management when acting as athletes’ supporters [37,40]. According to studies on higher education, educators understand how their role as instructors is critical in improving the quality of students’ education [41]. Educators must evaluate and examine themselves to clarify the goals they wish to achieve in their teaching and define their educational philosophy for “why” they are teaching [42]. In turn, clarification of the educational philosophy supports a sense of leadership [43]. As such, self-reflection may be implemented in higher education as a way for leaders to become aware of their own roles in terms of their educational philosophy [44]. Clarifying the educational philosophy reveals a sense of one’s role as a professional and, at the same time, eliminates the risk of distorting one’s role [45]. In college athletics, developing opportunities and programs for coaches to self-evaluate themselves as DC supporters of student athletes can foster appropriate competencies for coaches to support DCs rather than practicing coaching that is solely focused on competition. This may foster a balanced coaching competency. 

Beyond this, ASAEC was a higher important level of competency but with a large difference in terms of possession. Although the Japanese coaches, similarly to European DC supporters, recognized the need to be aware of the environment in which student athletes are engaged, there were significant differences between importance and possession. It is vital for coaches engaging in DC support to consider the diverse environmental and cultural backgrounds of student athletes [46,47]. Furthermore, coaches must be aware of the external issues and barriers that student athletes face in order to support their multifaceted development [13].

Additionally, coaches have to be knowledgeable in terms of understanding a student athlete’s environment from various perspectives—for example, awareness of changes in the athletic environment during the transition from their junior to their senior year [39,48,49], awareness of changes in the academic environment in the transition from secondary to higher education [50], and awareness of changes in the career environment in the transition to elite sports after graduation [51]. Therefore, it is necessary to create educational development programs that improve Japanese coaches’ competencies to quickly recognize the environmental and cultural backgrounds of athletes and to support student athletes’ DCs.

Empowerment competency is a coach’s ability to increase student athletes’ competencies, including improving their ability to plan their athletic and academic balance. It is important for coaches to help student athletes avoid potential problems and barriers and to increase their competencies so that student athletes can proactively deal with DC tasks independently [4,39]. Defruyt et al. [27] found that coaches with higher EC were more capable of handling DC support when student athletes were in a new social environment in which they were separated from their families and began living in student housing. However, while EC is a competency that coaches who implement DC support for student athletes are expected to possess, in this study, as well as in European studies [13], there are significant differences between importance and possession of EC. Therefore, it is necessary to introduce programs for developing coaches’ abilities to assist student athletes in autonomously developing competencies that they can apply to maintain a balance between academics and athletics.

Regarding OC, the effect size was moderate compared to those of the other competencies, although there were differences in the levels of its importance and possession. Organizational competencies consist of competencies that correspond to the culture of the organization to which a coach belongs, such as the ability to act in accordance with the (educational) mission of the university. Acting in accordance with the mission and culture of the organization is an important aspect of DC support [9]. Similar to our findings, Defruyt et al. [13] conducted a study of DC supporters in Europe, and they indicated that OC was only a small difference between importance and possession. In other words, coaches understand the importance of working according to the organizational culture of the university for DC support of student athletes and are willing to further implement it. However, considering that the response rate for this study was approximately 50% (the survey was originally sent to 300 participants), we assume that the participants who responded already have OC and are more likely to have OC than the coaches who did not respond. Future studies must better control for that possibility.

In a key finding, we found that ACC had the greatest difference between importance and possession. In other words, coaches perceived the competency of networking and coordinating with all stakeholders as being important for the development of a DC environment for student athletes, including universities, athletic clubs, sports federations, and families. However, the participants in this study perceived that they did not possess ACC. Defruyt et al. [27] found that coaches with higher levels of ACC possession were better able to respond effectively in environments where parents and professors did not offer student athletes DC support. Coaches’ abilities to generate an optimal environment for student athletes in their DC journeys had the effect of improving their athletic performance and promoting their academic activities [52]. In Japan, the UNIVAS is in the process of preparing a competency development program for coaches for the DC support of student athletes. We believe that, as part of this effort, it is most advisable to develop and offer programs to enhance coaches’ abilities to network and coordinate with the relevant stakeholders for DC support.

The relationship between coaches’ employment status and possession scores indicate that outsourced coaches had significantly lower possession levels than those of university faculties for ACC, ASAEC, and RC. In other words, coaches who support athletes while carrying out academic duties as university faculty members have higher competencies in DC support than outsourced coaches regarding “relationships with people and organizations surrounding student athletes”, “awareness of the student athlete’s environment outside of sports, such as academics”, and “relationships with student athletes”. According to a study examining the advantages and disadvantages of outsourced instructors in higher education, there are problems with external instructors not being able to actively collaborate with universities, largely because universities may not properly monitor instructors, and their contract management might be insufficient [53]. In the case of collegiate athletics and DC support for student athletes, outsourced coaches may have difficulty in actively collaborating with universities in accordance with university policies due to inadequate contract management. Universities need to provide information and programs to help outsourced coaches organize an appropriate, cooperative DC support system in collegiate athletics. In addition, the UNIVAS should develop a template DC support program for outsourced coaches to enhance DC support for student athletes in Japan.

In addition, we found a positive correlation between years of coaching experience and all DC competencies. Oshige et al. [54] examined the relationship between the years of nursing experience and professional competencies and found that those with more years of nursing experience tended to have greater competencies when it came to dealing with problems. In other words, the more years of experience in a particular profession, the higher the competency for dealing with various problems that occur in the occupation. We found that coaches with more years of coaching experience with student athletes had higher competencies for DC support, which is similar to the results suggested in previous studies. We believe that further research will identify what coaches with more years of coaching experience have done to develop their competencies in DC support for student athletes, thereby creating a chance to develop a program for coaches with less coaching experience. A qualitative study, such as one based on interviews, is also helpful in determining student athletes’ experiences and acquired competencies.

We identify several limitations of this study and suggest potential future studies on coaches’ competencies for DC support of student athletes. First, since this study was the first study to provide data measuring coaches’ competencies related to DC support in collegiate athletics in Japan, we were unable to compare our findings to similar research conditions in Japan. Therefore, there were limitations in interpreting the quantitative findings in relation to the existing qualitative DC studies. In the future, we need to conduct qualitative DC studies, such as interviews, as well. In addition, we need to investigate how the qualities and behaviors of coaches who support DCs affect the student athletes’ own perceptions and behaviors. In further studies, we need to conduct a longitudinal study to focus on the changes in coaches’ competencies and student athletes’ perceptions and behaviors toward DC implementation. Finally, the internal consistency of the scale items (Cronbach’s alpha coefficient) in this study was slightly higher than the criterion; thus, there is the potential for issues with each item on the DCCQ-J. Therefore, we need to conduct an additional study in Japan with a larger sample size and develop a measurement scale for DC support more appropriate to the Japanese situation.

## 5. Conclusions

In conclusion, we provided foundational data for the creation of DC support development programs for coaches. As a key takeaway, we found that the largest gap between importance and possession concerns ACC. Accordingly, we suggested that development programs in Japan for furthering coaches’ DC competencies should focus on enhancing their ACC. In addition, we found that there were differences in competencies’ possession scores, depending on the employment status of the coaches. Outsourced coaches were significantly lower in their level of possession than the university faculty. Furthermore, we found a positive correlation between coaching experience and all DC competencies. Coaches with more experience coaching student athletes were more likely to have higher competencies related to DC support.

The DCCQ-J is useful in coach development programs for DC support to evaluate the specific needs of particular target groups by surveying participants before and after the development program. The latter survey is effective in evaluating the success of DC support development programs for coaches in a systematic and evidence-based way.

## Figures and Tables

**Table 1:** Participant demographics.

Characteristics	*Mean (SD)*	*Range*
Age	43.15 (12.07)	23–68
Years of coaching history	12.83 (9.72)	2–39
	*n*	*Percentage (%)*
Gender		
Female	19	12.5
Male	133	87.5
Employment status of coaching		
University faculty	66	43.4
Outsourced coach	56	36.9
University staff	30	19.7
Types of sports		
Baseball	19	12.5
Soccer	15	9.9
Rugby	13	8.6
Swimming	11	7.2
Track and field	10	6.6
Volleyball	9	5.9
Basketball	8	5.3
Kendo	8	5.3
Archery	7	4.6
Handball	6	3.9
Gymnastics	4	2.6
Table tennis	4	2.6
Karate	4	2.6
Dance	4	2.6
Cycling	4	2.6
Tennis	3	2.0
Aikido	3	2.0
Wrestling	3	2.0
Judo	2	1.3
American football	2	1.3
Skiing	2	1.3
Ice hockey	2	1.3
Yachting	1	0.7
Badminton	1	0.7
Sailing	1	0.7
Sumo	1	0.7
Fencing	1	0.7
Triathlon	1	0.7
Golf	1	0.7
Lacrosse	1	0.7
Boxing	1	0.7

**Table 2:** Competency items for English to Japanese translation.

Factor	English (Original)	Japanese
ACC1	Ability to collaborate with key stakeholders (e.g., coach or parents) in the student-athlete’s life	学生アスリートの人生において、保護者、コーチなどのキーパーソンとなりうる人たちと協働することができる
ACC2	Ability to negotiate with DC stakeholders (e.g., student athletes, coaches, and teachers) ensuring that the interests of all are considered in the integration of a compatible outcome	デュアルキャリア教育においてキーパーソンたち（学生アスリート本人、コーチ、ゼミの担当教員、キャリアセンターの職員など）と交渉し、すべての人たちが納得できるような対応をすることができる
ACC3	Ability to build and coordinate a network of partners	学生アスリートのためのデュアルキャリア環境を整えるために、関係各所とネットワークを構築して調整する能力がある
ACC4	Ability to collaborate with decision-making bodies advocating for interests of student athletes	学生アスリートのデュアルキャリアに関する興味関心を促進するために、学生教育に関する意思決定を行う組織・会議体（部署）と協働する能力がある
ACC5	Sensitivity to environmental contexts (e.g., federation, family) that student athletes belong to	学生アスリートが所属する大学やクラブ、スポーツ連盟、家庭などの環境について様々な角度から、その状況の変化を感じ取ることができる能力がある
RSC1	Ability to reflect on own values and functioning to improve your practice	自身の学生アスリートに対する実践を向上させるため、自分の価値や役割を振り返る能力がある
RSC2	Ability to adapt the way of providing support in accordance to the feedback of others	他者からのフィードバックに応じて学生アスリートに対するサポートの方法を適切に修正する能力がある
RSC3	Ability to maintain own well-being and energy level necessary for work with student athletes	学生アスリートを育成するために必要な自身の心身の健康と指導に対する熱意を維持する能力がある
RSC4	Commitment to keep (self-) developing as a DC support provider	学生アスリートのデュアルキャリアを支援する立場にある者として、自身が成長し続ける能力がある
RSC5	Ability to realistically monitor and evaluate the effectiveness of your practice	自身の学生アスリートに対する実践の効果を客観的に捉え、評価する能力がある
OC1	Ability to complete administrative tasks (e.g., mails, data processing, file maintenance, etc.)	日々の管理業務をこなす能力（例：メールのやり取り、データ処理、書類管理等）がある
OC2	Ability to manage a variety of tasks (from one area to another) on a daily basis	日々の複数の仕事を同時並行で遂行する能力がある
OC3	Ability to be flexible in responding to unexpected events (e.g., injury) in the student-athlete’s life	学生アスリートにおける怪我などの不測の事態に柔軟に対応する能力がある（責任者としての調整能力）
OC4	Ability to coordinate different events in an effective manner	様々なスポーツイベント（試合、地域活動、大学イベントなど）を効果的に実施できるようにコーディネートする能力がある
OC5	Ability to act in congruence with the mission of the organization	大学のミッション（教育的使命）に沿って行動する能力がある
ASAEC1	Knowledge of the sports related to student athletes you work with	指導する学生アスリートに関するスポーツの知識がある
ASAEC2	Knowledge of the educational system(s)	大学の教育システムに関する知識がある
ASAEC3	Understanding the key transition phases of student athletes linked to the long term athlete development pathway	学生アスリートの長期的な成長につながる、重要な移行段階を理解している （例：1年生～4年生での役割の移行段階や競技引退前後の移行段階）
ASAEC4	Ability to take into account the diverse background (e.g., sociodemographic) of the student-athlete	学生アスリートの多様な社会的背景を理解し、配慮する能力がある（ジェンダー、宗教、人種など）
ASAEC5	Ability to take a holistic view of the student-athlete’s life	学生アスリート本人の人生を（競技成績だけでなく）包括的にとらえる能力がある
EC1	Ability to enhance athlete’s competencies concerning organization and planning of the student athlete’s life	学生アスリート自身が学生生活の構成バランス（競技と学業の両立等）を考えたり、計画を立てる能力を向上させることができる
EC2	Ability to make student athletes self-aware of their DC competencies	学生アスリートにデュアルキャリア*に関する能力を自覚させることができる
EC3	Ability to stimulate autonomy in student athletes	学生アスリートの自主性を促す能力がある
EC4	Ability to prepare student athletes for the challenges of specific transitions	学生アスリートが直面する特定の移行期の危機（競技引退など）に備えをさせる能力がある
EC5	Ability to enhance communication skills in student athletes	学生アスリートのコミュニケーションスキルを高める能力がある
EC6	Ability to make student athletes aware of the importance of rest and recuperation	学生アスリートに休息と回復の重要性を認識させる能力がある
RC1	Ability to refer the student athlete to another professional if necessary	学生アスリートに対し、必要に応じて他の専門家を紹介する能力がある
RC2	Ability to support student athletes emotionally in the face of setbacks	学生アスリートが挫折に直面した際、精神的なサポートをする能力がある
RC3	Ability to maintain a trust-based relationship with student athletes	学生アスリートとの信頼関係を維持する能力がある
RC4	Ability to treat each student athlete in an individualized manner	個人に合わせて、一人ひとりの学生アスリートを大切にする能力がある
RC5	Ability to conduct in-depth interviews for analysing the different steps of his/her life path	学生アスリートの人生における様々な重要な段階において、綿密に面談を行う能力がある
RC6	Ability to be an active and supportive listener	学生アスリートにとって、積極的かつ支援的な聞き手となる能力がある
RC7	Ability to maintain clear expectations and boundaries in the student athlete–support provider relationship	学生アスリートの成長をサポートする立場の関係性において、彼らへの明確な期待と適切な距離感を維持する能力がある

Notes: Advocacy and cooperation competencies, ACC; Reflection and self-management competencies, RSC; Organizational competencies, OC; Awareness of student athletes’ environment competencies, ASAEC; Empowerment competencies, EC; and Relationship competencies, RC.

**Table 3:** The validation factor analysis of DCCQ-J.

Competencies	Importance		Possession	
	*Loadings (* *λ)*	*α*	*Loadings (* *λ)*	*α*
ACC1	0.69	0.89	0.69	0.89
ACC2	0.77		0.77	
ACC3	0.77		0.88	
ACC4	0.84		0.82	
ACC5	0.79		0.77	
RSC1	0.68	0.84	0.75	0.86
RSC2	0.84		0.75	
RSC3	0.69		0.73	
RSC4	0.60		0.77	
RSC5	0.74		0.72	
OC1	0.51	0.76	0.49	0.81
OC2	0.57		0.53	
OC3	0.79		0.79	
OC4	0.57		0.67	
OC5	0.72		0.56	
ASAEC1	0.45	0.80	0.47	0.77
ASAEC2	0.55		0.57	
ASAEC3	0.71		0.55	
ASAEC4	0.82		0.69	
ASAEC5	0.86		0.87	
EC1	0.80	0.90	0.79	0.89
EC2	0.75		0.68	
EC3	0.79		0.79	
EC4	0.71		0.73	
EC5	0.78		0.73	
EC6	0.79		0.79	
RC1	0.84	0.89	0.73	0.89
RC2	0.77		0.73	
RC3	0.71		0.78	
RC4	0.74		0.72	
RC5	0.81		0.73	
RC6	0.84		0.79	
RC7	0.56		0.62	

Notes: Competencies are presented in the order of their competency factor and separated by a horizontal line. From top to bottom: Advocacy and cooperation competencies, Reflection and self-management competencies, Organizational competencies, Awareness of student athletes’ environment, Empowerment competencies, and Relationship competencies. A Loadings = Factor loadings for the importance and the possession of competencies within their competency factor.

**Table 4:** Perceived importance, possession, and difference (importance–possession) of the six DCSP competency factors.

Competency Factors	Importance		Possession		Difference		
	*M*	*SD*	*M*	*SD*	*M*	*SD*	*Cohen’s d*
Advocacy and cooperation competencies	4.18	0.68	2.91	0.61	1.27	0.65	1.95
Reflection and self-management competencies	4.46	0.52	3.94	0.64	0.51	0.57	0.89
Organizational competencies	4.23	0.57	3.92	0.62	0.31	0.63	0.50
Awareness of student athletes’ environment	4.38	0.54	3.91	0.58	0.47	0.59	0.80
Empowerment competencies	4.38	0.58	3.78	0.68	0.60	0.64	0.94
Relationship competencies	4.46	0.54	3.99	0.65	0.40	0.61	0.76

**Table 5:** Relationship between the employment status of coaches and their possession scores.

Variable	University Faculties	University Staffs	Volunteer Coaches	*F*	*p*	Post Hoc
	*Mean (SD)*	*Mean (SD)*	*Mean (SD)*			
ACC	3.02 (0.57)	2.99 (0.63)	2.74 (0.62)	3.57	0.05 *	3 < 1 *
RSC	4.04 (0.56)	4.04 (0.69)	3.79 (0.69)	2.62	n.s.	n.s.
OC	4.05 (0.63)	3.74 (0.61)	3.83 (0.59)	3.23	0.05 *	2 < 1 *
ASAE	4.06 (0.51)	3.74 (0.59)	3.79 (0.61)	4.79	0.01 **	2,3 < 1 *
EC	3.92 (0.57)	3.74 (0.65)	3.64 (0.80)	2.65	n.s.	n.s.
RC	4.15 (0.57)	3.92 (0.69)	3.84 (0.69)	3.54	0.05 *	3 < 1 *

Note: ** *p* < 0.01 and * *p* < 0.05. Advocacy and cooperation competencies, ACC; Reflection and self-management competencies, RSC; Organizational competencies, OC; Awareness of student athletes’ environment competencies, ASAEC; Empowerment competencies, EC; and Relationship competencies, RC.

**Table 6:** Relationship between the years of coaching and DC competencies’ possession.

	ACC	RSC	OC	ASAE	EC	RC
Years of coaching	0.33 **	0.34 **	0.33 **	0.45 **	0.34 **	0.33 **
Number of coaching seminars attended	0.20 *	0.14	0.18 *	0.10	0.11	0.07

Note: ** *p* < 0.01 and * *p* < 0.05. Advocacy and cooperation competencies, ACC; Reflection and self-management competencies, RSC; Organizational competencies, OC; Awareness of student athletes’ environment competencies. ASAEC; Empowerment competencies, EC; and Relationship competencies, RC.

## Data Availability

Not applicable.
